# Sleep Disorders in Neurodegenerative Diseases with Dementia: A Comprehensive Review

**DOI:** 10.3390/jcm14197119

**Published:** 2025-10-09

**Authors:** Natalia Siwecka, Michał Golberg, Dominika Świerczewska, Beata Filipek, Karolina Pendrasik, Adrianna Bączek-Grzegorzewska, Mariusz Stasiołek, Mariola Świderek-Matysiak

**Affiliations:** 1 Department of Neurology, Medical University of Lodz, 22 Kopcinskiego Street, 90-153 Lodz, Poland; natalia.siwecka@stud.umed.lodz.pl (N.S.); dominika.swierczewska@stud.umed.lodz.pl (D.Ś.); beata.filipek@stud.umed.lodz.pl (B.F.); karolina.pendrasik@stud.umed.lodz.pl (K.P.); adrianna.baczek@stud.umed.lodz.pl (A.B.-G.); mariola.swiderek-matysiak@umed.lodz.pl (M.Ś.-M.); 2 Department of Clinical Chemistry and Biochemistry, Medical University of Lodz, 5 Mazowiecka Street, 92-215 Lodz, Poland; 3 Department of Child Psychiatry, Medical University of Warsaw, 63A Zwirki i Wigury Street, 02-091 Warsaw, Poland; michal.golberg@umed.lodz.pl; 4 Department of Histology and Embryology, Medical University of Lodz, 7/9 Zeligowskiego Street, 90-752 Lodz, Poland; 5 Department of Microbiology and Pharmaceutical Biochemistry, Medical University of Lodz, 5 Mazowiecka Street, 92-215 Lodz, Poland

**Keywords:** sleep disorders, dementia, neurodegeneration, Alzheimer’s disease, Parkinson’s disease, neuropsychiatry, circadian rhythm, neuroinflammation, cognitive decline, integrated care

## Abstract

Dementia is a growing problem of global relevance, currently affecting over 55 million people worldwide. The number of new dementia cases is still increasing, primarily due to the aging of society. Dementia is defined as a substantial decline in cognitive function, and it is inherently associated with neurodegenerative diseases such as Alzheimer’s disease, Parkinson’s disease, dementia with Lewy bodies, frontotemporal dementia, and vascular dementia. Of note, most patients suffering from neurodegenerative conditions, in addition to cognitive impairment, often experience various types of sleep disorders, including insomnia, rapid eye movement sleep behavior disorder, sleep-disordered breathing, and circadian rhythm disturbances. There is increasing evidence of a bidirectional interaction between sleep disturbances and mental health. Disrupted sleep may directly aggravate neuropsychiatric symptoms, like depression, anxiety, agitation, and hallucinations, and conversely, such symptoms can make sleeping more difficult. This creates a feedback loop that inevitably leads to disease progression and deterioration in quality of life. In this review, we provide an up-to-date overview of the nature and mechanisms behind sleep disorders in major neurodegenerative diseases, summarize treatment strategies for handling sleep disturbances, and discuss the clinical relevance of sleep–mental health interactions in the context of neurodegeneration-associated dementia. Neurodegeneration is a complex problem on the border between neurology and psychiatry, and it poses a challenge to the healthcare system, as it requires multidisciplinary approaches for optimal management. Understanding the connection between sleep and neuropsychiatric symptoms offers further opportunities for better symptom control, improved quality of life, and slower cognitive decline.

## 1. Introduction

As life expectancy is constantly increasing, the number of people suffering from dementia and neurodegenerative diseases is rising steadily. Dementia is prevalent, as it is currently reported in up to 7% of individuals over 65 years old, and it is slightly higher in developed countries [1]. Additionally, there is an increasing incidence of young-onset dementia with a female predominance [2]. Neurodegenerative diseases pose challenges for healthcare systems, caregivers, and patients’ families. The main risk factors for developing dementia and related neurodegenerative conditions are advancing age, genetic profile, systemic vascular disease, and lifestyle [3]. Up to 40% of people with dementia have sleep disturbances and poorer disease prognosis as a result [4]. Among them, the most frequent is sleep-disordered breathing (SDB), followed by excessive daytime sleepiness (EDS), restless legs syndrome (RLS), rapid eye movement (REM) behavior disorder (RBD), and insomnia [5]. Neuropsychiatric symptoms are the main feature of all neurodegenerative disorders, with behavioral and psychological symptoms affecting nearly all dementia patients [6]. Apathy is the most frequent symptom, followed by depression, agitation, and aggression [7]. They arise in the moderate stages of disease and intensify as the disease progresses. Often, they appear before cognitive impairment [8,9]. Sleep disturbances increase the risk of mental health symptoms and the time of their onset [10]. An association of obstructive sleep apnea (OSA) with the risk for Alzheimer’s disease (AD) was shown, whereas insomnia was correlated with increased risk for both AD and vascular dementia (VaD) [11]. Both too long and too short sleep duration (≤4 or ≥10 h per night) contribute to an elevated risk of cognitive decline compared to moderate sleep length [12]. The inverted U-shaped association suggests that cognitive functions should be observed in middle-aged and older people susceptible to sleep issues [13]. Therefore, the goal of this review study is the following: (i) to describe clinical manifestations of sleep disturbances in different dementia-related neurodegenerative diseases; (ii) to review the molecular mechanisms underlying specific sleep disorders in these conditions; and (iii) to emphasize differences in sleep disturbances across neurodegenerative disease types that may support more accurate differential diagnoses and tailored therapeutic approaches.

## 2. Materials and Methods

This article represents a narrative review that focuses on the specific types of sleep disorders in neurodegenerative diseases, with an emphasis on the differences in their clinical presentation, molecular mechanisms underlying pathology, and clinical implications. This review format was selected because the scope of the study encompasses both clinical and preclinical findings, allowing for the integration of evidence from different methodological perspectives and disciplines. A systematic review design was considered unsuitable in this case, as it would limit the scope of sources and restrict the synthesis of both mechanistic and translational insights for sleep disorders in neurodegenerative diseases. A search of the literature was conducted for this study using PubMed, Scopus, Web of Science, and Google Scholar databases up to August 2025. The search strategy involved a combination of keywords such as “sleep disorders” or “sleep disturbances” with “dementia”, “neurodegeneration”, “Alzheimer’s disease”, “Parkinson’s disease”, “Lewy body dementia”, “frontotemporal dementia”, “REM sleep behavior disorder”, and related terms. Additional references were identified through manual screening of the bibliographies of key articles and recent reviews. The study design was not particularly restricted, and the inclusion criteria were broad, covering clinical studies, preclinical experiments, and review articles—this approach enabled providing a comprehensive and integrated overview of current knowledge on the link between neurodegeneration and sleep disorders. Articles that were not peer-reviewed, not written in English, or not directly relevant to the topic were excluded from the study. Once the suitable papers were selected, the evidence was synthesized qualitatively, with an emphasis on extracting similarities and differences in sleep-related manifestations across various neurodegenerative conditions, as well as identifying gaps and future research directions.

## 3. Pathophysiology of Neurodegenerative Diseases

Neurodegenerative diseases are a group of chronic, progressive disorders affecting the central nervous system (CNS). During neurodegeneration, nerve cells gradually lose their function and ultimately die, resulting in progressive brain dysfunction. This leads to the development of specific symptoms like cognitive decline or movement disorders, depending on which areas of the nervous system are affected by the neuronal loss [14]. As neurodegeneration is typically a slow, ongoing process, it is clinically presented as a gradual worsening of symptoms over time. For instance, it is estimated that in Parkinson’s disease (PD), up to 30–70% of neurons are already lost at the time of symptom onset [15]. Neurodegenerative disorders are usually characterized by a multifactorial etiology that involves a complex interplay of aging (a primary risk factor), genetic factors (inherited forms of disease), environmental exposures, and lifestyle choices (sporadic forms of disease) [16]. The key molecular mechanisms underlying neurodegeneration include the misfolding and aggregation of specific, pathogenic proteins (except for VaD, which is caused by vascular dysfunction and chronic hypoperfusion), mitochondrial dysfunction, oxidative stress, and neuroinflammation [17]. The mentioned processes create a toxic environment for neural cells that promotes further damage, accumulation of misfolded proteins, and neuronal network and synaptic dysfunction. There is no known cure for any neurodegenerative disease; however, current treatment options can help manage symptoms and improve quality of life [18]. The major pathological features of the most common neurodegenerative disorder types are listed in Table 1.

## 4. Sleep Disorders in Neurodegenerative Diseases with Dementia

### 4.1. Types of Common Sleep Disorders

Sleep is a fundamental physiological need, without which normal functioning is impossible. It consists of cyclic non-rapid eye movement (NREM) and REM phases. NREM occurs immediately upon falling asleep, characterized by normal muscle tone and reduced physiological activity (decreased blood pressure, heart rate, body temperature, and respiratory rate). The REM phase typically appears approximately 100 min after sleep onset and is associated with muscle twitches in the face and limbs. Sleep disorders are highly prevalent, affecting up to 50% of the general population and an even higher proportion of patients with dementia and related neurological diseases, such as AD, VaD, dementia with Lewy bodies (DLB), and PD. Emerging evidence from recent studies highlights the bidirectional relationship between sleep dysregulation and neurodegeneration, underscoring the potential for sleep interventions as modifiable risk factors. This section reviews the most common sleep disorders—insomnia, RBD, SDB, and circadian rhythm disturbances—in the context of dementia, drawing on the latest epidemiological, mechanistic, and clinical insights.

Insomnia is characterized by difficulty initiating or maintaining sleep, with the accompanying impairment of daytime activities, with symptoms occurring at least three times per week and persisting for at least three months. It affects up to 50% of older adults (aged 65+), with a higher prevalence in patients with dementia [37]. Recent meta-analysis indicates that insomnia significantly elevates the risk of all-cause dementia, particularly when co-occurring with major depressive disorder (MDD), which amplifies this association [11]. Neuropsychiatrically, insomnia symptoms such as difficulty initiating sleep (DIS) and sleep dissatisfaction are linked to a heightened risk of mild cognitive impairment (MCI) and poorer cognitive performance in older adults, including deficits in memory, attention, and executive function [38]. In the memory clinic setting, self-reported sleep disturbances correlate with objective polysomnographic findings, highlighting underdiagnosis and the need for integrated screening in dementia care [39].

RBD, involving dream enactment behaviors due to loss of REM atonia, serves as a prodromal marker for synucleinopathies, including MSA, DLB, and PD, with conversion rates exceeding 80% over a decade [40,41]. Isolated RBD contributes to the development of cognitive impairment and axial motor symptoms, such as gait instability and postural deficits [42]. A recent meta-analysis has found that patients with isolated RBD present impaired cognitive domains (specifically cognitive screening, memory, and executive functions), and the baseline presence of lower executive function or MCI has been pointed out as a risk factor for the conversion of RBD to a neurodegenerative disorder [43]. RBD exacerbates sleep fragmentation, leading to daytime somnolence, increased agitation, and behavioral disturbances in persons with dementia, such as frequent awakenings, reduced deep sleep, and heightened risk of neuropsychiatric symptoms like hallucinations or anxiety [44]. Reassessment of the role of amyloid-β (Aβ) in isolated RBD suggests that Lewy body pathology, rather than Aβ deposition, drives dementia risk, highlighting targeted neuroprotective strategies [45].

Another group is sleep-related breathing disorders, such as central sleep apnea, OSA, and sleep-related hypoventilation or hypoxia. In the general population, the prevalence of these conditions varies, ranging from 3% for OSA in women to 11% in men, with higher rates in older adults with dementia [46]. Central sleep apnea results from reduced activity of the respiratory center or impaired transmission of nerve impulses to the respiratory muscles. In contrast, the primary mechanism of obstructive sleep apnea involves excessive relaxation of the muscles maintaining airway patency. Both conditions lead to the cessation or shallowing of breath while sleeping. Symptoms of sleep-related breathing disorders include snoring, waking with a sensation of breathlessness, night sweats, or morning headaches. SDB is a modifiable risk factor for dementia, with prevalence rates over 50% in older patients with kidney failure or cognitive complaints [47]. One meta-analysis reveals that OSA increases the risk of all-cause dementia and its subtypes, particularly VaD [11]. Sex-specific analysis indicates higher dementia risks in women with known or suspected OSA over 10 years, underscoring gender differences in neuropsychiatric vulnerability, such as greater susceptibility to mood disorders and cognitive decline [48].

Circadian rhythm sleep–wake disorders affect approximately 30% of older adults [49]. These disorders involve a mismatch between the internal circadian rhythm and societal demands. Patients report insomnia at night and hypersomnia during the day. This group includes delayed sleep phase syndrome and irregular sleep–wake patterns. Differentiation of subtypes is possible through sleep diaries or actigraphy (when feasible). Circadian rhythm disturbances are integral to dementia pathophysiology, affecting up to 60% of patients, and they are linked to alterations in the suprachiasmatic nucleus (SCN), which is the brain’s primary circadian pacemaker [50]. Neuropsychiatrically, these disturbances contribute to sundowning, agitation, depressive symptoms, and increased caregiver burden, exacerbating overall cognitive and behavioral decline in dementia.

Sleep disorders are a common issue among patients with dementia, necessitating further research and exploration. The interplay between sleep disorders and dementia underscores the need for early screening and targeted interventions to mitigate cognitive and neuropsychiatric decline, ultimately improving quality of life for affected individuals and their caregivers.

### 4.2. Disease-Specific Sleep Profiles

Despite heterogeneous symptomatology and clinical course, patients with neurodegenerative conditions frequently experience sleep disturbances. The prevalence and type of sleep dysfunctions vary across neurodegenerative diseases, and the presence of specific sleep disorders might be used in differential diagnosis [51]. The main sleep disturbances described in patients with neurodegeneration include the following: (1) sleep–wake disorder: insomnia and EDS; (2) parasomnia: REM parasomnia (RBD and nightmares) and NREM parasomnia; (3) SDB; and (4) RLS [52]. The prevalence of the above-mentioned sleep disorders in selected neurodegenerative diseases is shown in Table 2.

AD is the most prevalent type of neurodegenerative disease, accounting for 60–80% of dementia cases and affecting approximately 10% individuals over age 65 [63,64]. It is estimated that 45% of AD patients experience sleep disturbances, often before the onset of cognitive symptoms [65]. The sleep concerns commonly seen in people with AD involve changes to the sleep–wake cycle. Disruption of the circadian rhythm contributes to insomnia (not sleeping at night), EDS (too much sleeping during the day), and sundowning, which manifests as increased anxiety and agitation in the late afternoon, that may last into the night and affect sleep [66]. Another characteristic feature of AD is intermittent nocturnal arousal, resulting in sleep fragmentation and reduction in total sleep time and sleep efficiency [67]. Decreased sleep duration, especially that of slow-wave sleep (SWS) and REM sleep, disrupts the process of memory consolidation in AD, which exacerbates the memory loss and cognitive decline [65]. Sleep loss may also worsen some neuropsychiatric symptoms, such as delusions and restlessness, which can in turn hinder the ability to fall asleep.

PD affects 1–2% of elderly people (>65 years old) living in high-income countries, and up to 80% of patients with PD suffer from sleep disturbances during the course of the disease [68]. Insomnia is characterized by difficulties in initiating sleep and maintaining sleep, and early morning awakening. Multiple factors contribute to insomnia in patients with PD. The most common problems are bradykinesia and rigidity, which make it difficult to turn over comfortably, while dystonia, painful leg cramps, and nocturia, accompanied by difficulty getting out of bed, are also significant factors that disrupt sleep quality. Anxiety and hallucinations are the main causes of difficulty in falling asleep in patients with PD [69]. EDS, presenting as somnolence and naps during the day, occurs in 21–76% of PD patients. Increased prevalence of EDS is associated with older age, PD duration, and akinetic-rigid phenotype of the disease [70]. Dopamine agonists, as well as some medications used to treat non-motor symptoms, such as antidepressants and antipsychotics, can cause EDS in patients with PD. The duration of the disease and the use of these medications also contribute to circadian rhythm disruption, as sleep begins in the evening and ends in the early morning. In about 20% of PD patients, RBD can develop before motor signs start [55]. RBD is associated with PD with old age, rapid progression of motor dysfunction, akinetic-rigid phenotype, freezing phenomenon, dyskinesias, EDS, and dementia [71,72]. OSA causes sleep fragmentation. In patients with PD, OSA is associated with age-related changes rather than disease-specific factors and, unlike in the general population, is not associated with body mass index (BMI) or gender, nor with disease duration or severity of Parkinsonian symptoms [73]. RLS, which tends to occur in the evening and during periods of inactivity, is characterized by a compulsion to move the legs. RLS in people with PD is clinically similar to idiopathic RLS; its incidence increases with disease duration and the use of dopaminergic medications in some patients [74,75].

**Figure 1:**
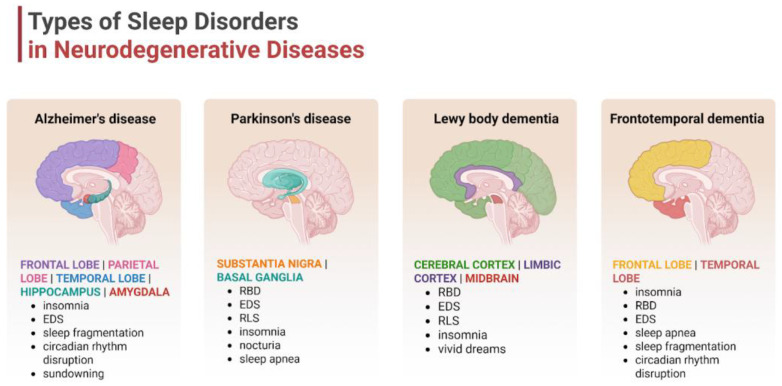
Types of sleep disturbances in the selected neurodegenerative disorders. Neurodegenerative diseases like Alzheimer’s disease, Parkinson’s disease, Lewy body dementia, or frontotemporal dementia are characterized by specific sleep patterns, which are directly associated with the affected brain regions. Abbreviations: EDS—excessive daytime sleepiness; RBD—rapid eye movement sleep behavior disorder; and RLS—restless leg syndrome. Created in BioRender. Siwecka, N. (2025) https://BioRender.com/cp49vwh.

Approximately 90% of people with DLB have at least one form of sleep disorder. Insomnia, EDS, and RBD are more common and more severe in people with DLB than in other dementias [59]. In patients with frontotemporal dementia (FTD), sleep is severely fragmented, with insomnia, EDS, and SBD frequently reported. REM behavior disorder is rare in patients with FTD [60]. Multiple system atrophy (MSA) is often associated with a range of sleep disorders, with the most common being RBD, followed by SDB, including stridor and OSA, with EDS observed in approximately 28% of patients [56,57,58]. Sleep disturbances occur in approximately 75% of patients with progressive supranuclear palsy (PSP) during the course of the disease and are associated with its duration. The most common are EDS, insomnia, and SDB [62,76]. In summary, in PSP and corticobasal degeneration (CBD), the incidence of RBD is significantly lower than in synucleinopathic PD, DLB, and MSA. VaD can significantly disrupt sleep patterns, leading to both insomnia and excessive daytime sleepiness. VaD is associated with an increased risk of developing cerebrovascular disease, and, particularly, OSA is correlated with an increased number of brain white-matter hyperintense lesions seen in small vessel disease [77] (Figure 1).

## 5. Mental Health Symptoms in Dementia

Behavioral and psychological manifestations of dementia are referred to as neuropsychiatric symptoms, and they accompany neurodegenerative diseases. This term refers to a broad spectrum of clinical presentations, including affective and psychotic symptoms. Psychiatric presentations can significantly influence the trajectory of underlying disease and impact the overall well-being of individuals and their caregivers [78,79].

Depression is one of the most prevalent neuropsychiatric symptoms in individuals with AD, second only to apathy, and can significantly exacerbate disability in this group of patients. Diagnosing depression in the context of dementia presents a subset of unique challenges, as elderly patients may not always exhibit the diagnostic criteria for MDD (DSM-5; ICD-10/11) and may require a more comprehensive assessment approach, including a psychological diagnostic process. One of the most widely recognized tools remains the Cornell Scale for Depression in Dementia (CSDD), which rates patients’ behavior in four categories. The Geriatric Depression Scale (GDS) is not a dedicated tool for dementia patients, but it might be used among elderly patients with signs of depressive episodes in general.

Anxiety disorders are frequently comorbid with dementia and can manifest as generalized anxiety disorder (GAD), panic attacks, or social anxiety. What is more, clinical observations have shown that anxiety at the age of 40–65 and older was associated with increased risk of all-cause dementia, especially AD and VaD [80].

Agitation or aggression in patients with symptomatic dementia is characterized by a spectrum of behaviors, including verbal outbursts and physical aggression. The mentioned behaviors are challenging for caregivers, contributing to their distress and early burnout. In some cases, aggressive behaviors may require long-term pharmacological management [81]. Clinical assessment may require additional high-sensitivity testing, including validated tools like the Spanish Neuropsychiatric Inventory (NPI), the Neurobehavioral Rating Scale (NBRS), and the Pittsburgh Agitation Scale (PAS) [82].

Psychotic symptoms such as delusions, hallucinations, or paranoia can also emerge in dementia, adding another layer of complexity to the clinical picture. They can be identified with a frequency of 34–63% and have the most diverse etiologies [83]. Depending on etiology, symptoms vary in frequency and presentation, with delusions often being persecutory or related to misidentification, while hallucinations are typically visual [83,84].

Apathy is defined as a reduction in emotional expression or goal-directed behavior. It is a common and often debilitating symptom in dementia patients. It may be associated with signs of depression and may contribute to social withdrawal, reduced self-care, and impaired cognitive function. Although clinical presentation may be similar, it is essential to distinguish apathy and depression, given their different implications for prognosis and management [85].

Early identification, appropriate classification, and differential diagnosis are crucial at the primary care level for the timely management of common age-related problems [78]. The heterogeneity of accompanying symptoms in dementia underscores the importance of individualized psychiatric assessment and treatment strategies that address the specific needs of each patient.

## 6. Mechanistic Links Between Sleep Disturbance and Neuropsychiatric Symptoms

### 6.1. Neuroanatomical Overlap

**Figure 2:**
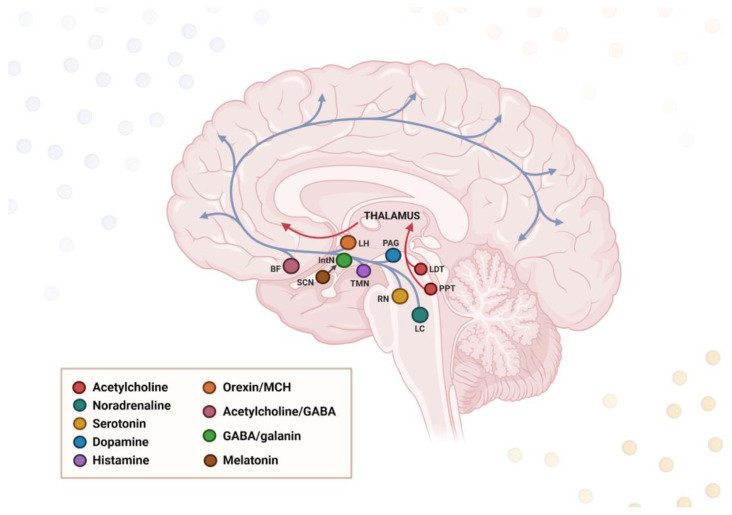
Neural circuitry governing wakefulness and sleep. The ascending reticular arousal system (ARAS) induces waking and alertness, whereas the hypothalamic intermediate nucleus (IntN) inhibits ARAS and thus promotes sleep. Both systems are under the influence of the suprachiasmatic nucleus (SCN), which regulates their activation levels and the switch between sleep and wakefulness. ARAS projections originate in the brainstem and extend to the cerebral cortex via the thalamus and hypothalamus. The legend presents the location of neural cell bodies for the specific neurotransmitters. LH—lateral hypothalamus; BF—basal forebrain; PAG—periaqueductal gray matter; TMN—tuberomammillary nucleus; RN—raphe nuclei; LDT—laterodorsal tegmental nucleus; PPN—pedunculopontine nucleus. Created in BioRender. Siwecka, N. (2025) https://BioRender.com/6fni0e0.

The transition between sleep and wake states is orchestrated by neuromodulatory centers located in the brainstem, basal forebrain, and hypothalamus. The latter contains the central circadian pacemaker, the SCN, which plays a critical role in adjusting the sleep–wake rhythm. The other key sleep-regulating regions sited in the hypothalamus involve the tuberomammillary nucleus (TMN) and the orexinergic and melanin-concentrating hormone (MCH) neurons in the lateral hypothalamus (LH) [86]. All above-mentioned brain areas consist of an interconnected network of nuclei, which, although limited to minor subcortical regions, modulate neuronal activity and cortical synchronization via vast projections throughout the brain; these extend to thalamic, hippocampal, and cortical areas [87]. The physiological sleep–wake rhythm is maintained by a complex interplay of mutual interconnections between various regions in the human brain. In general, different neural circuits control and orchestrate wake–sleep phases by the activity of wake-promoting, NREM-promoting, and REM-promoting cell populations. These populations interact via a “flip-flop switch” under the influence of SCN and determine the arousal state based on a mutual inhibition [88]. The excitatory wake-promoting neurons involved in an arousal state are spread across a number of nuclei throughout the brainstem, basal forebrain, and hypothalamus, referred to as the ascending reticular activating system (ARAS) [89]. More specifically, the activating system resides in the LH, locus coeruleus (LC), raphe nuclei (RN), and periaqueductal gray matter (PAG), which contain monoaminergic neuron populations [90]. By contrast, the sleep-promoting neurons in the hypothalamic intermediate nucleus (IntN) drive the sleep state primarily via inhibition of the wake-promoting neurons, depending on gamma-aminobutyric acid (GABA) neurotransmission [91]. In addition, the sleep–wake cycle regulation is fine-tuned by a complicated interplay of various external factors like neurotransmitters, hormones, and genetics, which will be discussed in more detail in the subsequent sections (Figure 2).

Many sleep-regulating brain areas are vulnerable to deposition of abnormal protein aggregates present in neurodegenerative conditions, and degeneration of these regions corresponds with specific sleep abnormalities. For instance, loss of MCH-synthesizing neurons at LH results in dysregulation of sleep–wake states [92], loss of galanin-expressing neurons in the IntN leads to sleep fragmentation [93], SCN degeneration causes sundowning effect [94], and lesions along the activating system (posterior LH, midbrain) induce EDS [95]. RBD is primarily caused by degeneration of noradrenergic neurons in the LC and cholinergic neurons in the brainstem, basal forebrain, and pedunculopontine nucleus (PPN), whereas loss of serotonergic neurons in RN is known to reduce the SWS phase [96]. 

A link between hypothalamic circuit dysfunction and sleep impairment in AD has been widely documented, and hypothalamic dysfunction is regarded as a key driver of sleep dysfunction in AD. Most sleep disturbances characteristic of AD, like low sleep efficiency, increased night waking, and sleep–wake disruption, are attributed to impaired connection between the SCN and the pineal gland [97]. On the other hand, disruption of hypothalamic projections from the SCN to the ventromedial nucleus (VMH), which regulates both daily rhythm and aggressive behaviors [98], was suggested as a potential mechanism of sundowning in AD. Postmortem studies revealed that sleep–wake rhythm alterations in AD patients were related to hypothalamic region atrophy [99], in particular SCN, LH (containing orexin neurons), and IntN (containing galanin neurons) [100]. In line with these findings, in AD brain specimens, both Aβ and tau pathology have been found in the hypothalamus [99]. The dysfunction of hypothalamic activity has also been observed in vivo in AD patients, in which the connections between the hypothalamus and limbic system were lost, and this correlated with nocturnal sleep disturbance [86]. It has also been suggested that sleep dysfunction in APOEε4 mutation carriers may be linked to early structural hypothalamic changes before the AD onset [101,102]. Among other brain regions, disruption of brain stem nuclei that are critical for sleep regulation, like dorsal RN, LC, ventral tegmental area, and laterodorsal tegmentum, might contribute to fragmented sleep, REM sleep deficits, and increased wakefulness, and these areas may be affected in AD [103]. For instance, LC takes part in the sleep–wake cycle regulation via complex anatomical connections with the hypothalamus, and this part of the brain also undergoes degeneration at early stages of AD [104,105]. In one of the MRI studies, patients with AD showed morphological changes in the brainstem (most prominent in dorsal RN) and midbrain, which may underlie erratic sleep patterns [106]. A separate study demonstrated that sleep deficiency (lower SWS and REM) was associated with inferior parietal region atrophy in early AD individuals [107]. Interestingly, it was suggested that the tau pathology in the brainstem might be, in fact, the trigger of sleep–wake dysregulation, as it was found that sleep issues in AD patients precede the deposition of Aβ plaques [108].

Similar to AD, PSP represents a form of tauopathy, but, regarding the sleep disturbance profile, PSP is primarily associated with severe insomnia. In patients with either AD or PSP, dysregulation of sleep–wake homeostasis was correlated with loss of the specific subcortical, wake-promoting neurons—orexinergic at LH, histaminergic at TMN, and noradrenergic at LC [109]. In a separate postmortem study of both AD and PSP brains, substantial neuropathological changes were detected in the IntN, with alterations being more pronounced in PSP (mainly a significant reduction in galanin NREM-promoting neurons) [110]. Some other specific sleep disturbances characteristic of PSP (reduced sleep efficiency, decreased REM sleep, and EDS) were proposed to result from dysfunction in sleep–wake regulating centers in the brainstem and hypothalamus [109].

RBD, a common phenomenon seen in synucleinopathies like PD, may arise from damage to the substantia nigra (SN) [111] and sleep-related brainstem nuclei in the rostral pontine tegmentum, such as PPN, LC, the dorsal and caudal RN, subcoeruleus nucleus (SubC), and the nucleus magnocellularis (NM). A recent study on RBD patients has found impaired structural connectivity in as many as 14 brainstem nuclei [112]. Specifically, damage to the pontomesencephalic tegmentum is thought to directly affect the REM phase, as this region contains neurons that promote REM sleep and muscle atonia by inhibiting cranial nerves and spinal cord motor neurons [113]. In line with this, the manifestation of RBD in PD patients was linked to more severe or extensive pathology in the brainstem [114]. On the molecular level, Lewy body pathology and neuronal loss can be found in this area in RBD, as well as in PD and DLB [115,116]. In the context of PD, the most common synucleinopathy, it is speculated that the accumulation and spread of α-synuclein occurs in the premotor period of the disease, affecting the sleep centers in the medulla and pons (LC, PPN, and SubC) before reaching the SN; this results in RBD symptoms before developing Parkinsonism [117]. In PD patients with RBD, it has been found that pontine sublaterodorsal tegmentum neurons expressing corticotropin-releasing hormone-binding protein (CRHBP) are largely reduced and contain pathologic α-synuclein [118]. PD patients with RBD, in addition to pontomesencephalic tegmentum atrophy, also demonstrated loss of volumes in other regions responsible for sleep–wake state regulation and motor activity: reticular formation, thalamus, hypothalamus, amygdala, putamen, and anterior cingulate cortex [119]. Other postmortem analyses of PD brain specimens revealed degeneration and α-synuclein pathology of the serotonergic neurons in the median RN [120], basal forebrain cholinergic nuclei (which causes RBD) [121], nigrostriatal dopaminergic pathways (related to EDS and RBD) [122,123], noradrenergic LC (leading to RBD) [123], and paramamillary and posterior nuclei of the hypothalamus [124]. In vivo, poor sleep quality in PD patients was attributed to parietal cortical thinning and brainstem atrophy [125]. EDS in PD individuals was associated with an increased surface area of the right anterior insula, contraction of the right putamen, and expansion of the right amygdala [126]. Other important regions affected in EDS include the ARAS components, as well as the caudate and putamen [127]. The external factors for EDS development in PD patients involve nocturnal sleep disturbances and the use of dopaminergic medication [128]. As regards RLS in PD patients, it has been related to increased gray matter volume in the posterior cingulate cortex and decreased functional connectivity in the sensorimotor network [129]. 

As neurodegeneration in DLB affects numerous neurotransmitter systems (acetylcholine, dopamine, and noradrenaline), multiple networks (both cortical and subcortical), and sleep-related structures (brainstem, forebrain, hypothalamus, thalamus, and midbrain), it is not surprising that sleep and arousal are heavily dysregulated in DLB. In a case report of a DLB patient, the specific clinical profile with mainly EDS symptoms was attributed to the degeneration of the hypothalamus, SN, and LC [95]. EDS in DLB patients was also, based on autopsy studies, attributed to degeneration of the nucleus basalis of Meynert (nBM) [130], and Lewy body pathology in neocortical and subcortical regions [131]. In both DLB and MSA, destruction of dopaminergic ventral PAG neurons was suggested to contribute to EDS [132], whereas sleep-disordered breathing may result from degeneration of brainstem respiratory nuclei, such as the pre-Bötzinger complex, nucleus raphe pallidus, and nucleus raphe obscurus, which are implicated in respiratory chemosensitivity and rhythmogenesis [133]. In DLB individuals with RBD symptoms, early brainstem involvement is observed, extending to the forebrain structures [134]. It was speculated that RBD in DLB might be related to reduced metabolism in several regions of the cholinergic system (namely, dorsolateral and medial frontal areas, bilateral superior parietal lobule, left precuneus, rolandic operculum, and amygdala) [135]. Loss of neurons with α-synuclein accumulation in the LC, SN, PPN, and laterodorsal tegmental nuclei was also pointed out as a core of RBD development in the course of DLB [25]. Recently, the metabolic activity of the left pulvinar of the thalamus in DLB was also suggested to have an effect on sleep time and sleep-related activity [59].

Concerning FTD, it remains disputable whether insomnia or circadian rhythm alterations are structural or behavioral, as sleep disturbances in these patients were associated with normal body temperature variation (suggesting an intact SCN). On the other hand, separate studies have pointed to a loss of basal forebrain and hippocampus neurons as a possible cause of altered circadian rhythm in FTD patients [136]. In genetic FTD, cortical thinning in frontal and parietal regions, as well as reduced volumes of sleep-relevant hypothalamic subunits, were also connected to greater sleep disturbances [137]. Moreover, increased sleep duration in FTD was linked to reduced cortical thickness in frontal regions [138].

Collectively, numerous data suggest that it is the affected brain region that determines the profile of sleep disturbances in dementia patients rather than the specific type of neurodegenerative process. Of note, the circadian clock SCN, in addition to controlling sleep–wake cycles, also plays a major role in mood regulation, both of which deteriorate during the progression of dementia [139]. LC degeneration has also been proposed as a culprit for the development of both arousal and mood symptoms (specifically depressive) due to noradrenergic dysfunction [140]. Disruption to thalamo-cortical rhythms was also shown to drive mood instability and switch from the state of quiet alertness to drowsiness, waking dreams, and agitation [141]. Importantly, sleep deprivation may contribute to reduced fronto-limbic connectivity, which provides implications for the development of neuropsychiatric symptoms like depression, apathy, agitation, or even psychosis in dementia patients [142,143]. This indicates the presence of a significant neuroanatomical overlap between sleep and behavioral disorders seen in dementia, which share common neural pathways and mechanisms.

### 6.2. Neurochemical Dysregulation

It is well-established that both neurotransmitters and neurosteroids are implicated in various neurophysiological processes, including sleep regulation. Such neurotransmitters as acetylcholine, dopamine, serotonin, histamine, noradrenaline, and orexin are believed to increase wakefulness and alertness, and a reduction in their levels may lead to EDS, a characteristic feature of neurodegenerative diseases [144]. Specific neural cell populations are dispersed throughout the brain as the ARAS components, namely LC (noradrenaline), dorsal and median RN (serotonin), ventral tegmental area and PAG (dopamine), PPN and laterodorsal tegmentum (acetylcholine), and TMN and posterior hypothalamus (histamine). These centers remain under the influence of orexin produced in the LH [96]. REM sleep is induced in particular by the brainstem’s cholinergic, GABAergic, and glutamatergic neurons, with the cell bodies located in the pontomesencephalic tegmentum (so-called REM-on neurons), and also by dopaminergic signaling at the ventral tegmental area [145,146]. NREM is promoted by the noradrenergic afferents from the LC and serotonergic afferents from the RN (REM-off neurons), and is characterized by high activity of GABAergic neurons in the basal forebrain and anterior hypothalamus [147]. Sleep phases are additionally mediated by GABAergic interneurons located in various brain regions, including the cortex, basal ganglia, and LC [148]. The proper balance between wake- and sleep-promoting circuits is maintained based on a mutual inhibition, according to a “flip–flop switch model”, which creates a self-reinforcing loop and provides transition between sleep and wakefulness [88].

Dysregulation of REM circuits at any level may lead to the development of sleep disturbances, especially RBD. As cholinergic pathways play a key regulatory role in activating the REM phase, reduced cholinergic transmission, which occurs in AD, could lead to decreased REM sleep duration [149]. The other important aspect of AD is serotonergic system damage, which results in altered mood and sleep–wake cycle deterioration [150]. In addition, it has been found that the degree of fractal activity disruption in AD patients is strongly associated with vasopressinergic and neurotensinergic neurons in SCN, which holds implications for circadian disturbances [151]. It has also been suggested that sundowning in AD patients may result from dysfunction of the histaminergic neurons in the hypothalamic TMN [152]. In PD, loss of dopaminergic transmission is associated with the dysregulation of essential clock genes responsible for circadian rhythm maintenance [153]. Dopamine imbalance in PD-specific regions was also linked to RBD and EDS [154]. The other studies have revealed that sleep dysfunction in PD is also related to a reduced level of serotonin [155,156], melatonin, glutamine, and acetylcholine levels at specific timepoints [156]. Moreover, it was found that medications used in PD treatment, like dopamine agonists or amantadine, contribute to EDS development [157]. As in AD, the process of ongoing cholinergic degeneration is also a characteristic feature of DLB. Loss of noradrenergic LC and dopaminergic SN neurons leads to a severe dysregulation of the REM circuits in DLB [158]. The sleep disturbances in FTD may also be attributed to altered levels of several neurotransmitters, like serotonin, dopamine, GABA, and mainly glutamate [159].

Recently, the role of orexin has been increasingly investigated in the context of neurodegeneration. Orexin is a neuropeptide produced in the LH with a critical role in regulating alertness, and loss of orexin neurons is associated with narcolepsy. In contrast to wake-active orexin, hypothalamic orexin neurons also express MCH, which promotes sleep and suppresses wakefulness by inhibiting monoaminergic systems [160]. It has been found that a significant loss of orexin-producing neurons occurs in AD and PD [154]. In FTD, orexin dysregulation was associated with impulsive behavior, hedonism, and excessive alcohol consumption [161]. Melatonin is a monoamine hormone, synthesized primarily in the pineal gland from serotonin. It is regarded as a key regulator of the circadian rhythm, and it additionally demonstrated antioxidant and neuroprotective properties by decreasing the accumulation of pathogenic proteins like Aβ [162]. Melatonin has also been shown to improve cholinergic and glutamatergic signaling [163,164]. It has been found that the secretion of melatonin gradually decreases with both aging and the progression of neurodegenerative disorders, mainly AD and PD. Low levels of melatonin, in addition to disrupting circadian rhythm, have been associated with sundowning syndrome and other types of sleep disturbances [154].

In addition, sleep disturbances may induce neurotrophin levels to decline, which in turn affects neuron survival, synaptic plasticity, memory, and sleep [165]. One of microglia’s functions during sleep is the elimination of weak synapses and synapse remodeling [166]. It was reported that sleep affects reduced dendritic spine number and increased synapse formation, which confirms the diurnal fluctuation of the synaptic network. In particular, REM sleep is believed to maintain homeostasis in the CNS, and REM disturbances might affect neuronal excitability, integrity, neurogenesis, synaptic formation, memory consolidation, and ultimately drive neurodegeneration [167].

### 6.3. Neuroinflammation and Stress

**Figure 3:**
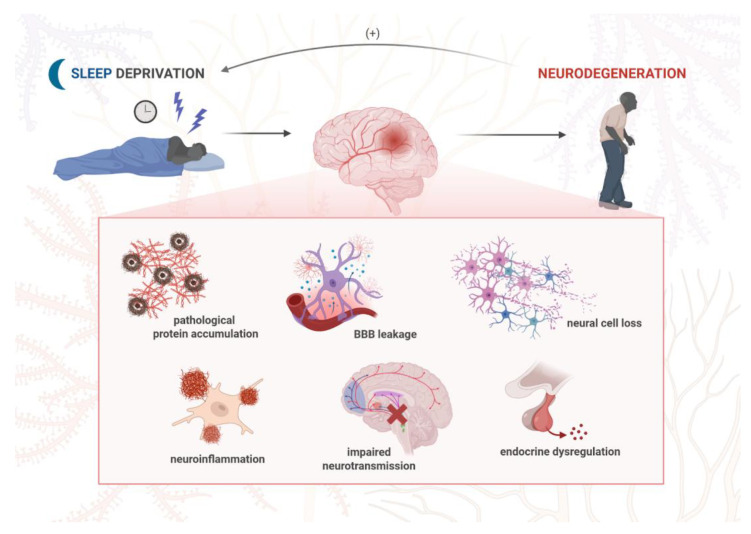
The molecular mechanisms underlying the effect of sleep deprivation on neurodegeneration development. Chronic sleep disturbances are associated with a number of neuropathological events, including neuroinflammatory response, increased accumulation of pathogenic proteins, loss of BBB integrity, neural cell death, disrupted neurotransmission, and hormone signaling. Such changes at the molecular level not only drive neurodegeneration and dementia symptoms but also aggravate sleep disturbances, which potentiate the sequence of pathological events in the nervous system in a positive feedback loop. Created in BioRender. Siwecka, N. (2025) https://BioRender.com/q495vp2.

There is a reciprocal interaction between stress exposure and sleep disturbances. While both acute and chronic stress lead to sleep disruption, which may worsen cognitive dysfunction and other neuropsychiatric symptoms, sleep deprivation itself is known to trigger an inflammatory process within the CNS. Neuroinflammation may, in turn, contribute to behavioral changes such as anxiety, hopelessness, or anhedonia by increasing the expression of specific inflammatory mediators [168]. The neuroinflammatory response is driven by non-neuronal cells known as glia. One type of glial cell, astrocytes, has an impact on sleep architecture by mediating glymphatic clearance and adenosinergic signaling (A1, A2, A3), and both these processes can be disrupted by chronic stress [169]. Both sleep disturbances and stress conditions also trigger activation of microglia, which drive neuroimmune response by overproducing inflammatory cytokines: interleukin-1β (IL-1β), interleukin-6 (IL-6), tumor necrosis factor α (TNF-α), and NLR family pyrin domain containing 3 (NLRP3) inflammasome. When secreted into the microenvironment, the cytokines can influence other neurons and astrocytes and, in turn, cause neuroinflammation [170]. These molecules are excessively generated over the course of neurodegeneration, and such overactivated microglia are known to exacerbate neuronal damage and synaptic loss [171]. Furthermore, released inflammatory cytokines drive Aβ and tau aggregation, leading to neuronal dysfunction and death, and, clinically, the aggravation of neuropsychiatric and sleep-related symptoms [172]. Apart from the CNS, inflammatory molecules from the periphery can also alter sleep physiology. It has been found that a marker of systemic inflammation, C-reactive protein (CRP), at higher levels is associated with increased sleep fragmentation and risk of incident dementia [173]. In addition, the vagus nerve transmits inflammatory signals between the CNS and peripheral nervous system via the so-called gut–brain axis; this provides complex interactions between stress, sleep, and inflammatory responses [174]. It is also known that microbial metabolites may influence neural pathways. Furthermore, sleep disturbances were linked to impaired blood–brain barrier (BBB) permeability as a possible result of neuroinflammation [175]. Many of these effects converge on the disruption of synaptic processes, such as neurotransmitter balance, synaptic plasticity, and pruning, which, in turn, contribute to the pathophysiology of neurodegenerative and psychiatric disorders [176]. In rodents, sleep deprivation led to anxiety-like behaviors in the mechanism involving increased cortisol secretion, expression of clock genes, and several inflammatory cytokines [168,177], as well as learning and memory deficits due to increased levels of IL-6 and microglia activation in the hippocampus [178]. Conversely, long sleep may be a consequence of neuroimmune reaction and a marker of ongoing neurodegeneration. Individuals at increased risk of AD onset presented a hypersomnia sleep profile and higher cerebrospinal fluid (CSF), including neuroinflammatory marker IL-6 and monocyte chemoattractant protein 1 (MCP-1) [179,180]. Moreover, low levels of IL-6 were associated with both elevated risk for AD and increased OSA severity, but the underlying mechanism is not yet understood [181]. Both systemic and neuroinflammation are considered early events in the course of AD, prior to Aβ pathology [182], and they are known to increase Aβ burden [183], thereby accelerating AD progression and cognitive decline. On the other hand, it is believed that NREM sleep facilitates clearance of extracellular Aβ, as neuroinflammation is negatively correlated with the NREM sleep duration [184]. Short sleep duration in AD patients was linked to neuroinflammation, increased orexin levels, Aβ deposition, and significant cognitive impairment. In AD mice, sleep disruption, astroglial activation in the hippocampus, and elevated hypothalamic orexin levels preceded Aβ neuropathology and cognitive impairment [185]. Some of the key effects of sleep disruption are increased levels of reactive oxygen/nitrogen species (ROS/RNS) and the accumulation of protein aggregates, such as Aβ and α-synuclein [186]. A possible mechanism of protein plaque clearance is its autophagic degradation through endo-lysosomal pathways [187] or via the glymphatic drainage system. The glymphatic system is particularly active during noradrenergic-mediated NREM sleep, and it is responsible for clearing out metabolic and protein waste products. Its activity decreases with aging and due to the degeneration of the LC area. The inclusions of α-synuclein within the LC were observed in prodromal PD and linked to sleeping disorders and impaired glymphatic activity, both of which drive neuroinflammation [188]. PD mice exhibited significant sleep architecture changes, spectral shifts in electroencephalogram (EEG), and neuroinflammatory features such as microglial activation and astrogliosis in the key sleep-regulating regions [189]. Patients with RBD presented increased microglial activation in the SN along with reduced dopaminergic function in the putamen [190]. Daily variations in the level of S100β, the main astrocytes’ product, were associated with the severity of PD and perceived sleep disruption [191]. Sleep deprivation in PD mice exacerbated loss of dopaminergic neurons and motor deficits in the mechanism involving gut microbiota-associated microglial activation and oxidative stress [192]. It has also been suggested that the brain-derived neurotrophic factor/tropomyosin receptor kinase B (BDNF/TrkB) pathway is associated with sleep disturbances in this model [193] (Figure 3).

### 6.4. Circadian Misalignment

The hypothalamic SCN constitutes the central biological clock of the circadian system, as it controls the production of the chronobiotic hormone melatonin in the pineal gland. These activities are synchronized by environmental light detected by the retina of the eye, followed by transmission via the retinohypothalamic tract to the SCN and then through the pineal pathway [194]. The activity of the central circadian pacemaker is regulated by melatonin in a negative feedback loop and the expression of clock genes, namely, circadian locomotor output cycles protein kaput (CLOCK), period (PER), cryptochrome (CRY), and brain and muscle ARNT-like protein 1 (BMAL1) [195]. The SCN, in addition to sleep–wake rhythm, coordinates various other time-specific activities, including metabolism, endocrine function (especially corticosteroid secretion), and immune reactions. Clock genes like BMAL1 can regulate the secretion of cytokines and chemokines, thereby affecting glial cell activation in the CNS [196], and expression of these genes declines with senescence [197]. While the major role of melatonin is the regulation of circadian rhythms and thus duration and quality of sleep, it also exerts antioxidant and neuroprotective properties. Thus, melatonin deficiency may not only be a consequence of neurodegeneration but also contribute to its development. Circadian disorders are defined as disturbances in the sleep–wake cycle and are a common phenomenon in the course of aging, becoming even more pronounced in neurodegeneration. It has been demonstrated by multiple studies that circadian dysregulation affects the severity of mental impairment and that it occurs in the early, preclinical stages of various types of dementia [198,199]. Specifically, in AD, disruptions in the sleep–wake cycle, like delirium, agitation, and sleep–wake disturbance, are attributed to disrupted melatonin production and rhythms, especially decreased nocturnal melatonin levels [200] and decreased melatonin CSF levels [201]. It is believed that these changes are driven by degeneration of the SCN, resulting in dysfunctional sympathetic control of pineal melatonin synthesis. In particular, loss of neurons expressing vasopressin, vasoactive intestinal peptide (VIP), and melatonin receptor type 1 in the SCN has been found in postmortem examinations of AD cases [202]. Interestingly, while SCN of AD patients shows typical alterations, like pretangles and tangles, such neuropathological hallmarks characteristic of AD have not been detected in the pineal glands [52]. The other possible pathological mechanisms of circadian dysfunction in AD involve dysregulation of circadian rhythm of β1-adrenergic receptor mRNA [203], which regulates SCN innervation to the pineal gland, depletion of serotonin (a melatonin precursor), increased monoamine oxidase A (MAO-A) activity [202], and dysregulated CLOCK mRNA expression [204]. It has also been suggested that the effect of environmental light on the circadian system in AD individuals may be disrupted, as a specific variant close to the melatonin receptor type 1A gene (MTNR1A) is associated with increased risk for both Alzheimer’s disease and intolerance to shift work [205]. It has been hypothesized that diurnal oscillations in Aβ levels could be controlled by the circadian timing system [206]. Such diurnal fluctuations of both motor and nonmotor symptoms are also highly prevalent in PD patients, suggesting the role of the circadian system in the process of dopaminergic transmission. A number of studies showed mixed results regarding melatonin rhythm changes in PD, most pointing to decreased melatonin secretion in PD patients [207]. However, increased salivary melatonin levels during the day were linked to dysregulation of systemic circadian rhythms underlying EDS in PD [208,209]. An increase in serum melatonin level was also found in PD patients at advanced stages, suffering from dyskinesias [210]. Dopaminergic treatment, normally applied to mitigate Parkinsonian symptoms, was shown to affect circadian rhythms [211] via an increase in secretion of melatonin [212]. This points to a possible interaction between dopamine and melatonin activity and a crosstalk between dopaminergic transmission and the circadian clock. On the molecular level, a decreased level of melatonin receptors (MT1 and MT2) was found in the SN of PD patients, which may account for circadian abnormalities due to disruption of dopaminergic pathways [213]. The other finding in PD histopathological exams is the SCN atrophy, which may explain decreased core body temperature and altered hormonal secretion, including cortisol and melatonin, as well as dysregulated blood pressure rhythms [214]. The endocrine dysregulation was confirmed by separate studies that found elevated plasma cortisol levels in PD patients [215]. The data regarding other neurodegenerative disorders is limited. To date, one study demonstrated the disruption of melatonin secretion and clock gene BMAL1 expression in DLB patients [216]. Some studies have found that FTD is also associated with rest–activity rhythm alterations, but the underlying molecular mechanisms remain unknown [138,217]. Importantly, there is ample evidence that diurnal and circadian changes in dopaminergic neurotransmission influence mood-related behavior as well as addiction-related behavior, which is the case for PD and DLB [218]. 

## 7. Bidirectional Relationship: Sleep and Mental Health in Dementia

A cross-talk between mental health and sleep in dementia may be described as a complex interplay that can significantly impact the course and management of the disease, while disrupted sleep and wake balance affects 70% of patients in early-stage dementia [219].

One of the most substantial psychiatric symptoms, anxiety, in the general population, can profoundly disrupt sleep patterns, leading to insomnia and fragmented sleep architecture. Anxiety usually manifests as rumination and excessive worry, which can lead to sleep disturbances. Imbalanced sleep patterns, in turn, will exacerbate neuropsychiatric symptoms, especially in dementia-affected patients, who may already be burdened with psychiatric symptoms. Sleep loss may contribute to worsening hallucinations, agitation, and mood disturbances [220,221]. Moreover, behavioral disturbances are frequently reasons for acute hospitalization and long-term institutional placement, which may exacerbate insomnia. Sleep–wake cycles during hospitalization may be disrupted, especially when sedative medications were required, so that the patient is neither continuously awake nor asleep during a 24 h period, which closes the loop [222].

A bidirectional relationship between the described phenomena supports the concept of a feedback loop, where sleep disturbances and mental health symptoms perpetuate and amplify each other. This may create a cycle that is difficult to break without appropriate management. Given the high prevalence, biological importance, and health implications associated with the disrupted balance of sleep in older individuals, there is a need to focus on this topic in order to improve patient outcomes and reduce the intensity of dementia-accompanying symptoms [223,224].

## 8. Clinical Assessment and Diagnosis

A comprehensive assessment, capable of characterizing sleep patterns among patients with dementia, is essential for neuropsychiatric evaluations. Sleep disturbances can significantly impact cognitive function, mood, and behavior, as described earlier in this review.

One of the most important aspects of clinical evaluation is the sleep history, obtained from both the patient and caregivers. By encompassing details about sleep duration, sleep quality, sleep timing, and any associated sleep-related behaviors, appropriate management can be incorporated in patients’ treatment to improve their sleep [225].

Due to cognitive impairments associated with dementia, there is a loss of necessary objective information about patients’ functioning. One of the tools able to provide the required data is actigraphy. This is a non-invasive method that tracks activity via a wrist-worn device, providing valuable insights into sleep–wake patterns. This type of device can be worn for several consecutive days, providing sleep data for patients in non-hospital care settings. Unfortunately, they are expensive, which limits their broad use [225,226].

Caregiver reports are essential in assessing sleep patterns in patients with cognitive impairment, and they remain the foundation for the identification and development of any care plan by offering useful information about night-time behaviors and sleep quality that the patient may not accurately report. The most basic tool—sleep diaries—can be kept; this is where a family member records the patient’s bedtime. Additionally, they can describe night-time awakenings or document their rise time. Identifying whether the patient has more difficulty falling asleep or staying asleep may help determine the most effective management approach [227].

For clinical assessment, validated sleep symptom rating scales are useful and beneficial for initial assessments, problem identification, and follow-ups to measure the effectiveness of treatments. A widely used questionnaire is the Sleep Disorders Inventory (SDI), which is specifically designed and validated for use with dementia patients, and is useful in both home settings and long-term care facilities. It simply characterizes the frequency, severity, and caregiver burden of sleep-disturbed behaviors over a two-week period. It includes difficulty falling asleep, night awakenings, and excessive daytime sleepiness [228].

For patients in an early dementia state, the Pittsburgh Sleep Quality Index (PSQI) can be used, as it is used by self-reporting. The questionnaire is used to evaluate sleep quality over a month. It includes 19 items organized into seven components, which together generate a single global score. Completing the measure typically takes 5 to 10 min [229].

When a patient or caregiver reports daytime sleepiness, the appropriate tool is the Epworth Sleepiness Scale (ESS). This instrument asks the individual or caregiver to rate the likelihood of falling asleep across eight typical situations, on a scale from 0 (never) to 3 (high chance). Questions like being stopped in traffic can be omitted if the person no longer drives. A total score of 10 or higher suggests the need for further assessment for common sleep disorders [225,230].

## 9. Therapeutic Approaches

### 9.1. Non-Pharmacological Interventions

Non-pharmacological methods administered in sleep disorders among dementia patients include cognitive behavioral therapy for insomnia, sleep hygiene, structured routines, and bright light therapy. Cognitive Behavioral Therapy for Insomnia (CBT-I) is a structured, evidence-based, non-pharmacological intervention designed to improve sleep quality. CBT-I aims to improve habits and behaviors that disrupt sleep by identifying them and modifying bedtime thoughts and routines. Standard therapy consists of six to eight sessions, which include two core components: Sleep Restriction Therapy (SRT), which involves limiting time in bed to increase sleep efficiency, and Stimulus Control Therapy (SCT), which involves avoiding any kind of wakeful activities in bed. Supplementary components are sleep hygiene (SH) and cognitive therapy (CT), which help identify and change irrational and negative thought patterns [4,231].

SH refers to specific behavioral and environmental practices established in the late 1970s to help individuals with insomnia. The recommended practices consist of the following: participating in regular physical activity; techniques for managing stress; reducing noise levels in the bedroom; and following a well-organized sleep routine, as well as avoiding the intake of caffeine, nicotine, alcohol, and daytime naps [232,233].

Structured routines and environmental cues are fundamental non-pharmacological strategies for treating insomnia. They assist in managing the sleep–wake cycle by promoting regular patterns and alleviating stress related to sleep. Environmental cues are physical or sensory signals in the bedroom and home environment, such as light, noise, temperature, and scent, that help the brain recognize sleep time. Enhancing these signals can lead to better sleep quality, fewer disturbances at night, and the reinforcement of healthy sleep–wake cycles, which are particularly crucial for elderly individuals experiencing insomnia or those suffering from dementia [234].

Therapy involving exposure to intense artificial light (typically 10,000 lux), also known as bright light therapy (BLT), helps in regulating the body’s circadian rhythm. Bright light therapy mimics natural sunlight to adjust the sleep–wake cycle. Specifically, this therapy is helpful for those with insomnia, delayed sleep phase syndrome, shift work sleep disorder, seasonal affective disorder (SAD), and sleep issues in dementia [235,236].

Non-pharmacological interventions are typically recommended as an initial strategy to address sleep disorders in dementia patients. Their advantages include minimizing drug interactions and limiting the side effects of medications. However, if these methods do not sufficiently resolve sleep problems, a wide range of pharmacological treatment options may be considered.

### 9.2. Pharmacological Strategies

A range of pharmacological agents has been investigated to alleviate sleep disturbances in dementia, yet their clinical use requires careful consideration of efficacy, safety, and the potential impact on cognition and metabolism. Medications employed to address sleep disturbances in individuals with dementia encompass atypical antipsychotics, benzodiazepines, and other GABA-modulating agents like zolpidem, zopiclone, and zaleplon, as well as melatonin, sedative antidepressants, and antihistamines [237].

Despite the frequent clinical use of sedative and antipsychotic agents in dementia, methodologically rigorous trials addressing sleep outcomes remain scarce. Most available studies rely on limited or indirect measures of nocturnal behavior, and randomized controlled trials with objective sleep assessments are virtually absent. Consequently, pharmacotherapy is not considered a first-line approach for insomnia or other sleep–wake disturbances in patients with dementia, given both the paucity of evidence and the heightened vulnerability of this population to adverse effects, such as confusion, daytime somnolence, or falls [238,239].

Due to the limited efficacy and significant safety concerns, Z-drugs (zopiclone, zaleplon, and zolpidem) in people with dementia should generally be avoided, with treatment decisions guided by the Beers criteria and a preference for non-pharmacological strategies whenever possible [240]. Among nonbenzodiazepines, eszopiclone was recently demonstrated to have a much lower addictive potential than the classical Z-drugs. When compared to other Z-drugs, which should be applied for a maximum of one month, eszopiclone can be used longer, for up to six months, with good safety and tolerability (especially in elderly patients) [241].

Clinical trials investigating melatonin supplementation in patients with AD-related dementia have yielded inconsistent findings. Across five randomized studies, doses up to 10 mg nightly did not lead to meaningful improvements in sleep outcomes, such as total sleep time or sleep efficiency [237,242,243,244,245,246]. Similarly, ramelteon, a selective melatonin receptor agonist, demonstrated no superiority over the placebo in a small cohort with mild-to-moderate dementia. Importantly, both agents appeared safe, with no reports of serious adverse events. These findings suggest that while melatonergic drugs are well tolerated, their clinical utility for sleep disturbance in dementia remains limited, and routine use cannot be recommended based on current evidence [237].

A common cause of sleep dysfunction in people with dementia is increased tension and anxiety, and there is an emphasis on the application of anti-anxiety medications in this population. Selective serotonin reuptake inhibitors (SSRIs) are a good choice in elderly patients with underlying medical conditions due to a good metabolic profile, with citalopram and escitalopram being preferred due to their additional selectivity towards 5-HT1A and 5-HT2 receptors [247]. Other antidepressants, particularly trazodone and doxepin, may provide short-term benefits for sleep in individuals with insomnia, but the lack of robust long-term data and the potential for significant side effects highlight the need for cautious use and further research. Among the sedating antidepressants, trazodone has been most frequently studied for insomnia in dementia. Evidence from a small randomized controlled trial (n = 30) suggests that a nightly dose of 50 mg may increase total sleep duration by approximately 40 min and improve sleep efficiency. The impact on nocturnal awakenings and wake time after sleep onset was less clear. Importantly, the drug was well tolerated over a two-week treatment period, without reports of serious side effects. While promising, these findings are limited by the short trial duration and small sample size, underscoring the need for larger, long-term studies to establish trazodone’s efficacy and safety in this population [237,248,249].

Orexin receptor antagonists, such as suvorexant and lemborexant, represent a novel therapeutic class targeting wake-promoting neuropeptides implicated in Alzheimer’s disease pathology. Two randomized controlled trials with over 300 participants demonstrated that these agents significantly improved sleep continuity, with average gains of nearly 30 min in total sleep time and reductions of 15 min in nocturnal wakefulness. Sleep efficiency also increased modestly. Side effects, including next-day somnolence or confusion, were not significantly more common than in placebo groups, suggesting acceptable short-term safety. Given their mechanism and clinical profile, orexin antagonists appear to hold the most promise among currently tested pharmacotherapies, though long-term data for dementia are still lacking [237,250,251].

Current evidence indicates that benzodiazepine receptor agonists can be effective for short-term treatment of insomnia, but their long-term safety and efficacy remain uncertain, and potential risks such as cognitive impairment, dependence, and rebound insomnia warrant cautious use.

The use of barbiturates and antipsychotics for insomnia lacks evidence of effectiveness and carries considerable risks, making them unsuitable for the management of chronic insomnia.

Over-the-counter antihistamines are widely used for insomnia, but their questionable efficacy and significant risk of adverse effects, especially in older adults, limit their role as a safe treatment option [238].

### 9.3. Multidisciplinary Management

Since a large proportion of insomnia cases stem from psychiatric disorders, while neurological conditions such as restless legs syndrome are also common causes, both psychiatric and neurological expertise are essential in its assessment. Effective diagnosis and management, therefore, rely on close collaboration between psychiatrists and neurologists [252].

Because of the multifactorial origins of sleep disturbances in dementia—including underlying neuropathology, impaired sleep regulation, coexisting primary sleep disorders, and medication side effects—effective diagnosis and management of sleep disturbances in this population require an integrated, multifactorial approach. Multicomponent interventions currently appear to hold the greatest promise [253,254].

Moreover, considering the bidirectional link between sleep and overall health, it is essential that a wide range of healthcare professionals—including pulmonologists, endocrinologists, cardiologists, otolaryngologists, psychologists, pediatricians, dentists, and primary care physicians—routinely assess sleep quality and duration as a part of their clinical practice [255].

## 10. Challenges and Future Directions

Current management of sleep disorders in dementia patients faces several challenges that need to be overcome in order to improve the clinical outcomes. First, sleep disturbances remain severely underdiagnosed in dementia patients, partially due to communication difficulties and symptom overlap. It may be challenging to differentiate between the neurodegeneration-associated mental health symptoms and those resulting from sleep abnormalities or other psychiatric comorbidities. Thus, such cases should be carefully evaluated by experienced professionals and might require the use of specialized clinical tools. Second, most studies on this topic are primarily cross-sectional, so they do not provide an answer to the following basic question: which came first, sleep disturbances or neuropsychiatric symptoms? In this regard, further longitudinal studies are needed to determine the causality of sleep–mental health problems clearly. Furthermore, effective management of patients with neurodegenerative disorders demands integration of care, which means collaboration between neurologists, psychiatrists, sleep specialists, and caregivers. Such multidisciplinary approaches encompassing various aspects of the disease should become a standard in routine dementia care. There is also a need for personalized interventions in terms of individualized treatment plans for patients with neurodegenerative conditions. Each approach should be tailored to the type of neurodegeneration, specific sleep disorder profile, and prevailing neuropsychiatric symptoms. This would allow for better symptom control, improved quality of life, and, possibly, slower progression of the disease, offering a therapeutic window, especially for patients with early- and mid-stage dementia.

There is an increasing role of innovative technologies like wearable devices and digital platforms, facilitating the monitoring of sleep and behavior patterns in real-time, which is particularly useful in the outpatient or home settings. The other emerging technology in sleep medicine is a non-invasive brain stimulation (NIBS), which represents a non-pharmacological approach to treat sleep disturbances. NIBS modulates sleep by targeting neural activity of the specific brain regions in a non-invasive manner; this provides a beneficial effect on either sleep quality or other neurological and psychiatric symptoms. While NIBS has been tested in insomnia patients with encouraging results [256], it also holds promise for treating more complex neurodegenerative disorders. In PD, NIBS techniques improved motor function [257] and cognition, and alleviated depressive symptoms [258], and they were also helpful in enhancing sleep quality and modifying sleep architecture [259]. A specific type of NIBS, repetitive transcranial magnetic stimulation (rTMS), was shown to increase cognitive performance in individuals with AD [260] and potentially affect sleep [261], but large-scale studies are needed to confirm this effect. Thus, NIBS could become an integrative approach for treating sleep disorders and other aspects of neurodegenerative conditions. Of note, given that sleep disorders occur relatively early in patients with neurodegeneration, it would be beneficial to make a diagnosis at prodromal phases; this could be achieved by the development of disease-specific biomarkers. Recently, p-tau217 has been identified as a reliable blood biomarker for AD [262], and the assay detecting α-synuclein in the CSF of PD patients has also been developed [263]. Further advances in the biomarker area are essential for early identification of both patients with neurodegenerative disease and those with sleep disorders in this population (e.g., via actigraphy), as it would facilitate the application of the appropriate early interventions.

Overall, a better understanding of the sleep–mental health interaction in neurodegenerative diseases opens up new research and clinical possibilities. Integration of sleep-focused assessment and treatment in dementia patients could improve the management of dementia-related neuropsychiatric symptoms.

## Figures and Tables

**Table 1:** The pathological hallmarks of major neurodegenerative diseases associated with dementia.

Disease	Pathogenic Protein Aggregates	Pathogenic Mutations	Affected Brain Regions	Affected Neurotransmitters	Clinical Symptoms
AD	Aβ (extracellular senile plaques), tau (intracellular NFT) [19]	APP, PSEN1, PSEN2, APOEε4 [19]	entorhinal cortex, hippocampus, temporal, parietal, and frontal lobes [20]	acetylcholine [21]	memory loss, executive dysfunction, confusion, language problems, and mood changes [20]
PD	α-synuclein (Lewy bodies, Lewy neurites) [22]	SNCA, PRKN, PINK1, DJ-1, LRRK2, GBA [19,23]	midbrain, basal ganglia [22]	dopamine [21]	Parkinsonism (tremor, bradykinesia, rigidity, and imbalance) [19]
DLB	α-synuclein (Lewy bodies, Lewy neurites) [22]	SNCA, GBA, APOEε4 [24]	basal ganglia, limbic system, brainstem, temporal, parietal, and frontal lobes [21]	acetylcholine, dopamine [21]	fluctuating cognition, Parkinsonism, visual hallucinations, and RBD [19,25]
FTD	tau, TDP-43, FUS [26]	C9orf72, MAPT, GRN [27]	frontal and temporal lobes [27]	serotonin, dopamine, glutamate, GABA [28]	behavioral and personality changes, and language difficulties [27]
MSA	α-synuclein (GCIs) [29]	SNCA, COQ2 [22]	basal ganglia, brainstem, cerebellum [29]	dopamine, serotonin, and acetylcholine [30]	Parkinsonism, autonomic dysfunction, ataxia, and pyramidal dysfunction [29]
PSP	4R tau (intracellular NFT, neuropil threads, tufted astrocytes, and oligodendroglial coiled bodies) [31]	MAPT, LRRK2, DCTN1 [31]	basal ganglia, brainstem [31]	various [28]	Parkinsonism, frequent falls, supranuclear gaze palsy, and bulbar dysfunction [31]
CBD	4R tau (astrocytic plaques, oligodendroglial coiled bodies) [32]	MAPT, GRN, C9orf72, PRNP [33]	cerebral cortex, and basal ganglia [32]	various [28]	asymmetric Parkinsonism, dystonia, apraxia, and sensory deficits [33]
VaD	-	NOTCH3, APOEε4, MTHFR [34]	cerebral cortex, white matter, basal ganglia, and thalamus [35]	acetylcholine, serotonin, and dopamine [36]	reduced mental speed, executive dysfunctions, confusion, memory loss, and language problems [35]

Abbreviations: AD: Alzheimer’s disease; PD: Parkinson’s disease; DLB: dementia with Lewy bodies; FTD: frontotemporal dementia; MSA: multiple system atrophy; PSP: progressive supranuclear palsy; CBD: corticobasal degeneration; VaD: vascular dementia; Aβ: amyloid-β; NFT: neurofibrillary tangles; GCIs: glial cytoplasmic inclusions; and RBD: rapid eye movement sleep behavior disorder.

**Table 2:** The prevalence of sleep disorders in selected neurodegenerative diseases (based on Shen et al.) [52].

Disease	Insomnia	EDS	RBD	Nightmares	SDB	RLS
PD [53,54,55]	32–44%	21–76%	39–46%	17–30%	27–48%	14%
MSA [56,57,58]	19%	28%	88%	-	Stridor 30–42% OSA 15–37%	4.7–28%
DLB [59]	26–75%	11–100%	76%	83%	34–60%	-
FTD [60]	48%	64%	Rare	Rare	68%	8%
CBD [61]	Rare	-	14%	-	Rare	Rare
PSP [62]	60%	60%	11–28%	-	55%	3.7–58%

Abbreviations: EDS: excessive daytime sleepiness; RBD: rapid eye movement (REM) sleep behavior disorder; SDB: sleep-disordered breathing; RLS: restless leg syndrome; PD: Parkinson’s disease; MSA: multiple system atrophy; DLB: dementia with Lewy bodies; FTD: frontotemporal dementia; CBD: corticobasal degeneration; PSP: progressive supranuclear palsy; and OSA: obstructive sleep apnea.

## Data Availability

Not applicable.

## Abbreviations

The following abbreviations are used in this manuscript:

SDB	Sleep-disordered breathing
EDS	Excessive daytime sleepiness
RLS	Restless legs syndrome
REM	Rapid eye movement
NREM	Non-rapid eye movement
RBD	REM sleep behavior disorder
SWS	Slow-wave sleep
OSA	Obstructive sleep apnea
BMI	Body mass index
AD	Alzheimer’s disease
PD	Parkinson’s disease
DLB	Dementia with Lewy bodies
FTD	Frontotemporal dementia
MSA	Multiple system atrophy
PSP	Progressive supranuclear palsy
CBD	Corticobasal degeneration
MDD	Major depressive disorder
DIS	Difficulty initiating sleep
MCI	Mild cognitive impairment
CSDD	Cornell Scale for Depression in Dementia
GDS	Geriatric Depression Scale
GAD	Generalized anxiety disorder
NPI	Neuropsychiatric inventory
NBRS	Neurobehavioral rating scale
PAS	Pittsburgh agitation scale
PSQI	Pittsburgh sleep quality index
SDI	Sleep disorders inventory
ESS	Epworth sleepiness scale
SCN	Suprachiasmatic nucleus
TMN	Tuberomammillary nucleus
MCH	Melanin-concentrating hormone
LH	Lateral hypothalamus
ARAS	Ascending reticular activating system
LC	Locus coeruleus
RN	Raphe nuclei
PAG	Periaqueductal gray matter
IntN	Intermediate nucleus
PPN	Pedunculopontine nucleus
VMH	Ventromedial nucleus
SN	Substantia nigra
SubC	Subcoeruleus nucleus
NM	Nucleus magnocellularis
CRHBP	Corticotropin-releasing hormone-binding protein
nBM	Nucleus basalis of Meynert
GABA	Gamma-aminobutyric acid
CNS	Central nervous system
CSF	Cerebrospinal fluid
BBB	Blood–brain barrier
EEG	Electroencephalogram
ROS	Reactive oxygen species
RN	Reactive nitrogen species
CRP	C-reactive protein
IL-1β	Interleukin-1β
IL-6	Interleukin-6
TNF-α	Tumor necrosis factor α
NLRP3	NLR family pyrin domain containing 3
MCP-1	Monocyte chemoattractant protein 1
BDNF	Brain-derived neurotrophic factor
TrkB	Tropomyosin receptor kinase B
CLOCK	Circadian locomotor output cycles protein kaput
PER	Period
CRY	Cryptochrome
BMAL1	Brain and muscle ARNT-like protein 1
VIP	Vasoactive intestinal peptide
MAO-A	Monoamine oxidase A
MTNR1	Melatonin receptor 1A gene
MT1	Melatonin receptor 1A
MT2	Melatonin receptor 1B
CBT-I	Cognitive behavioral therapy for insomnia
SRT	Sleep restriction therapy
SCT	Stimulus control therapy
SH	Sleep hygiene
CT	Cognitive therapy
BLT	Bright light therapy
SAD	Seasonal affective disorder
Aβ	Amyloid-β
SSRIs	Serotonin reuptake inhibitors
NIBS	Non-invasive brain stimulation
rTMS	Repetitive transcranial magnetic stimulation
